# Measuring quality of life in trials including patients on haemodialysis: methodological issues surrounding the use of the Kidney Disease Quality of Life Questionnaire

**DOI:** 10.1093/ndt/gfac170

**Published:** 2022-06-11

**Authors:** Hannah M Worboys, Nicola J Cooper, James O Burton, Hannah M L Young, Ghazala Waheed, James Fotheringham, Laura J Gray

**Affiliations:** Department of Health Sciences, University of Leicester, Leicester, UK; Department of Health Sciences, University of Leicester, Leicester, UK; Department of Cardiovascular Sciences, University of Leicester, Leicester, UK; Leicester Diabetes Centre, University of Hospitals of Leicester NHS Trust, Leicester, UK; Diabetes Research Centre, College of Life Sciences, University of Leicester, Leicester, UK; Department of Respiratory Sciences, College of Life Sciences, University of Leicester, Leicester, UK; Leicester Clinical Trials Unit, University of Leicester, Leicester, UK; School of Health and Related Research, University of Sheffield, Sheffield, UK; Department of Health Sciences, University of Leicester, Leicester, UK

**Keywords:** end-stage renal disease, haemodialysis, methods, quality of life

## Abstract

**Background:**

Haemodialysis (HD) treatment causes a significant decrease in quality of life (QoL). When enrolled in a clinical trial, some patients are lost prior to follow-up because they die or they receive a kidney transplant. It is unclear how these patients are dealt with in the analysis of QoL data. There are questions surrounding the consistency of how QoL measures are used, reported and analysed.

**Methods:**

A systematic search of electronic databases for trials measuring QoL in HD patients using any variation of the Kidney Disease Quality of Life (KDQoL) Questionnaire was conducted. The review was conducted in Covidence version 2. Quantitative analysis was conducted in Stata version 16.

**Results:**

We included 61 trials in the review, of which 82% reported dropouts. The methods to account for missing data due to dropouts include imputation (7%) and complete case analysis (72%). Few trials (7%) conducted a sensitivity analysis to assess the impact of missing data on the study results. Single imputation techniques were used, but are only valid under strong assumptions regarding the type and pattern of missingness. There was inconsistency in the reporting of the KDQoL, with many articles (70%) amending the validated questionnaires or reporting only statistically significant results.

**Conclusions:**

Missing data are not dealt with according to the missing data mechanism, which may lead to biased results. Inconsistency in the use of patient-reported outcome measures raises questions about the validity of these trials. Methodological issues in nephrology trials could be a contributing factor to why there are limited effective interventions to improve QoL in this patient group.

**PROSPERO Registration:**

CRD42020223869

## Graphical Abstract

**Graphical Abstract:**
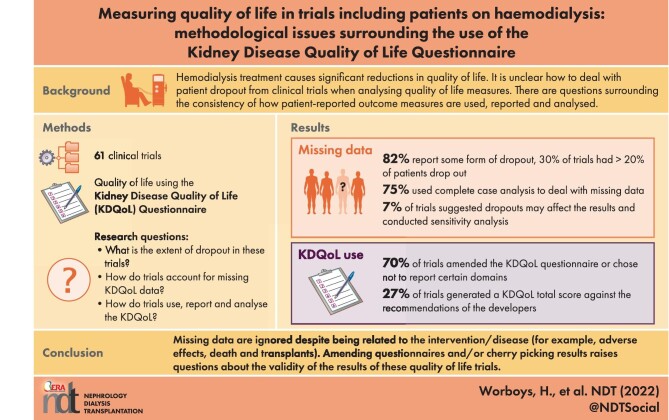

KEY LEARNING POINTS **What is already known about this subject?**

- A high treatment burden has led to a significant decrease in quality of life (QoL) in this patient group. The importance of research in this area is apparent and necessary in an attempt to improve the day-to-day lives of patients.
- There exists much literature about the poor quality of nephrology trials, including high levels of dropout in clinical trials and problems with recruitment and retention.
- Ignoring informative dropouts, i.e. dropouts related to the intervention or disease (e.g. adverse effects, death and transplantation) can lead to biased results, as the remaining sample is not a random subset of the original sample.

**What this study adds?**

- This is the first article to systematically assess the current practice of trials measuring QoL in dialysis patients.
- It looks specifically at the Kidney Disease Quality of Life Questionnaire, as this measure scores highly in psychometric properties and is good at capturing changes in QoL among these patients.
- It highlights the misuse of adequate statistical methods when dealing with missing data, the amendment of validated questionnaires and the tendency to make within-group comparisons, unadjusted analysis and not report a primary outcome. It also highlights that dropouts are ignored, despite the literature highlighting that this could lead to biased trial results.

**What impact this may have on practice or policy?**

- It hghlights to researchers and clinicians the need for better use of statistical concepts to ensure future research is of a better standard.
- A better research standard could change the results of future trials and lead to more interventions being approved for this patient group.
- This review forms the basis of future work that will look at appropriate ways to deal with patients in the primary data analysis who drop out of trials due to adverse effects, death and transplantations.

## INTRODUCTION

An estimated 800 000 people living in America rely on dialysis treatment for end-stage renal disease (ESRD) [1]. These patients have a significant treatment and symptom burden, greatly affecting their quality of life (QoL). QoL is defined by the World Health Organization as ‘an individual's perception of their position in life in the context of the culture and value systems in which they live and in relation to their goals, expectations, standards and concerns’ [2]. Low levels of QoL among these patients have led to an increasing number of clinical trials focusing on improvements in QoL. However, many trials conclude without being able to meaningfully improve QoL [3]. Existing literature highlights the poor methodological quality of nephrology trials [4], which could be contributing to the lack of meaningful results.

**Figure 1:**
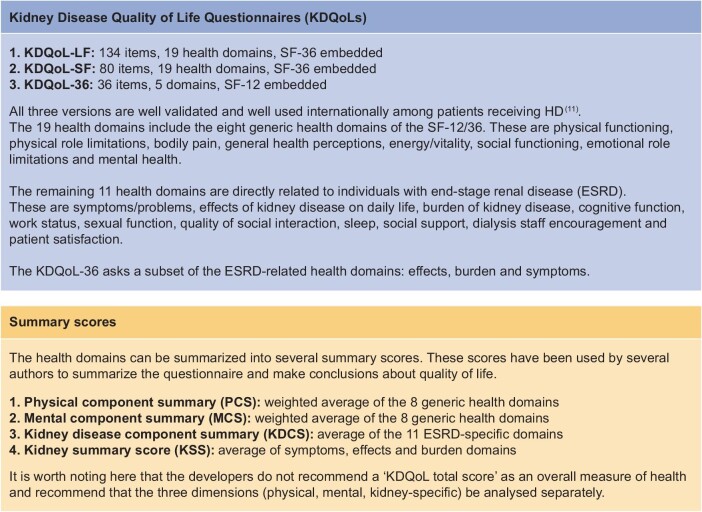
Details of KDQoL questionnaire.

Patient-reported outcome measures (PROMs) are key to assessing self-reported QoL. The Kidney Disease Quality of Life (KDQoL) Questionnaires are well-validated, reliable, condition-specific PROMs [5] designed to provide a comprehensive assessment of QoL among patients with ESRD and score highly in psychometric properties (consistency, validity and reliability). There are three versions of the questionnaire [6–8], which are described in Fig. 1. All versions of the KDQoL have the Short Form (SF)-12/SF-36 embedded in the questionnaire, a widely used instrument measuring two distinct components of QoL: physical and mental [9].

The KDQoL questionnaires have been validated in many patient populations [10, 11] to ensure that they accurately capture changes in the QoL of patients with ESRD when used in a clinical trial. It is important for validated questionnaires to be administered according to the specifications of their developers in order to retain the desired properties. This review looks at how closely the use of the KDQoL questionnaires aligns with the recommendations of the developers.

Missing KDQoL data is common in trials of haemodialysis (HD) patients, as relatively high proportions of patients either die or receive a transplant before completing the trial. Much literature exists discussing methods for dealing with missing data and the consequences of not doing so [12]. Previous reviews highlight the use of complete case analysis and single imputation methods to deal with missing QoL data [13]. However, these methods are only valid under strong assumptions about the missing data mechanism, i.e. whether they assume the missing QoL data to be missing completely at random (MCAR), missing at random (MAR) or missing not at random (MNAR) [14]. A detailed explanation of these concepts is included in Table 1 and Supplementary data, Appendix Table A1. Guidelines to impute and use complete case analysis are only valid if the missing data are random (i.e. MCAR or MAR) and unrelated to the treatment or intervention. Limited guidance exists on what to do otherwise and questions remain about how missing data are dealt with in practice.

**Table 1.** Differences between MCAR, MAR and MNAR mechanisms

Missing data mechanism, according to Rubin [14]	Assumption	Example
MCAR	Missing data and HRQoL outcome are independent	Participant moves abroad
	The reason for dropout is unrelated to the participants’ current health status	
MAR	Missing data/dropout depend on the observed longitudinal measurements	If male participants are less likely to report HRQoL data and dropout
	Dropouts related to baseline characteristics	
MNAR	Missing data/dropout depend on the unobserved longitudinal measurements	Dropout due to adverse effect, transplantation and death
	Directly related to the participant's current health status	
	Missing values cannot be modelled exclusively from the data of the observed participants	

We systematically reviewed published trials that measured QoL in HD patients using the KDQoL questionnaires to address the following questions: How do trials use, report and analyse the KDQoLs questionnaires? How do trials account for missing KDQoL data (specifically death/transplant) in their analysis?

## MATERIALS AND METHODS

This systematic review is reported in line with the Preferred Reporting Items for Systematic Reviews and Meta-Analysis (PRISMA) statement [15]. The PRISMA checklist is provided in Supplementary data, Appendix Table A2. The protocol for this review has been published elsewhere [16].

The search strategy was developed with the assistance of a specialist health sciences librarian and reviewed by a nephrologist. MEDLINE, Web of Science, Cochrane Central Register of Controlled Trials, Scopus and Cumulative Index of Nursing and Allied Health Literature were searched using combinations of keywords and topics. The original search strategy, developed in MEDLINE, is included in Supplementary data, Appendix Table A3. Databases were searched from inception to 16 November 2021. Searches were limited to publications available in English. Due to the methodological nature of the review, ongoing studies and unpublished trials were excluded.

### Inclusion and exclusion criteria

We included phase 3 clinical trials of any design measuring QoL using any version of the KDQoL questionnaire in adults (age ≥18 years) receiving HD. QoL could be a primary or secondary outcome.

We excluded trial protocols and reports of secondary analyses. We excluded trials that recruited a mix of patient treatments (HD, peritoneal dialysis and transplantation).

### Screening

The review was conducted using Covidence version 2 software. All searches were imported into Covidence. Duplicates were removed. Title and abstract screening was conducted independently by two reviewers (H.W. and G.W.). Full-text screening was conducted by three reviewers (H.W., G.W. and H.Y.). Each study was reviewed independently by at least two reviewers and any disagreements between reviewers were resolved by discussion.

### Data extraction

Data extraction was performed in Covidence using a predetermined extraction form. Pilot extraction was conducted on eight studies to amend and retest the extraction form. Two reviewers (H.W. and H.Y.) performed data extraction independently and any differences were resolved by consensus. Authors of trials with insufficient information to complete data extraction were contacted for further information.

### Analysis

The information extracted was exported and tabulated. The results were synthesized using descriptive statistics. The quantitative analysis was conducted in Stata/IC version 16.0 (StataCorp, College Station, TX, USA).

### Deviations from the protocol

Initial search strategies included both the KDQoL and SF-36 as measures of QoL. It was agreed by all authors that trials assessing QoL using the SF-36 could be omitted due to the number of trials found using the KDQoL (*n* = 399). This review is not aimed at analysing the intervention effects and focuses on the methodological quality of trials, therefore it was agreed a risk of bias assessment was unnecessary.

## RESULTS

### Study characteristics

**Figure 2:**
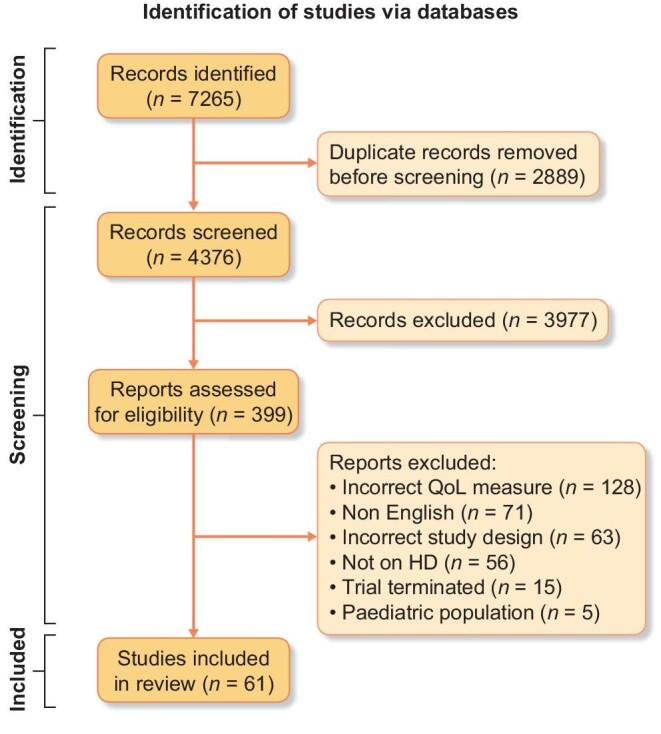
PRISMA flow diagram.

A PRISMA flow diagram detailing the identification of studies is displayed in Fig. 2. The number of articles identified for title and abstract screening was 4376. After the exclusion of the SF-36 articles, the number of articles meeting the inclusion and exclusion criteria was 399. The final review consisted of 61 trials. Throughout data extraction, 14 authors were contacted: 11 regarding their calculation of the KDQoL total score, 2 regarding their statistical analysis and 1 regarding the methods for dealing with missing data. Only one author responded to the e-mail.

The study characteristics for included studies are presented in Table 2. Most studies were randomized controlled trials [RCTs; 45 (74%)]. The remaining studies were quasi-RCTs [13 (21%)]; one trial of repeated measures within a single group, one trial of two active treatment arms without a control and one trial of multiple active treatment arms without a control. The trials reporting QoL as the primary outcome made up 33% of the total trials (*n* = 21). The breakdown between the three measures was 80% (*n* = 49) of trials used the KDQoL-SF, 13% (*n* = 8) used the KDQoL-36 and 7% (*n* = 4) used the KDQoL-LF. Most trials [42 (70%)] had a study duration of ≤6 months.

**Table 2.** Study characteristics

Study ID	Country	Year	Study design	RCT type	Multicentre	HRQoL primary	HRQoL instrument	KDQoL primary outcome	CONSORT	Baseline measurement	HRQoL measurements, *n*	3 months	3–6 months	6–12 months	>12 months
Atevik 2020	Turkey	2020	RCT	Two-arm parallel		✓	KDQoL-36	Not specific	✓	✓	2	✓			
Borzou 2020 [17]	Iran	2020	Quasi-RCT		✓	✓	KDQoL-SF	Not specific		✓	2	✓			
Chang 2016 [18]	Taiwan		Quasi-RCT			✓	KDQoL-36	Not specific		✓	2	✓			
Cukor 2014 [19]	USA	2014	RCT	Crossover			KDQoL		✓	✓	2	✓			
Dai 2020 [20]	China	2020	RCT	Two-arm parallel	✓		KDQoL-SF		✓		1			✓	
de Lima 2013 [21]	Brazil	2013	RCT	Multi-arm parallel			KDQoL-SF		✓	✓	2	✓			
deFreitas 2020 [22]	Brazil	2020	RCT	Two-arm parallel			KDQoL-SF		✓	✓	3			✓	
Deziel 2007 [23]	Canada	2007	RCT	Two-arm parallel			KDQoL-SF		✓	✓	2		✓		
Duarte 2009 [24]	Brazil	2009	RCT	Two-arm parallel			KDQoL-SF		✓	✓	3			✓	
Feldt-Rasmussen 2006 [25]	Multi- country	2007	RCT	Multi-arm parallel	✓		KDQoL-SF		✓	✓	1				
Figueiredo 2018 [26]	Brazil	2018	RCT	Factorial			KDQoL-SF		✓	✓	2	✓			
Fitschen 2017 [27]	USA	2017	RCT	Two-arm parallel			KDQoL		✓	✓	2		✓		
Foley 2009 [28]	Multi-country	2005	Multi-arm active treatments		✓		KDQoL			✓	6				✓
Fukuda 2015 [29]	Japan	2015	RCT	Two-arm parallel			KDQoL-SF		✓	✓	3	✓			
Greenwood 2021 [30]	UK	2021	RCT	Two-arm parallel	✓	✓	KDQoL-SF	KDQoL PCS	✓	✓	2		✓		
Habibzadeh 2020 [31]	Iran	2019	RCT	Multi-arm parallel			KDQoL-SF		✓	✓	2	✓			
Heo 2016 [32]	South Korea	2016	Quasi-RCT			✓	KDQoL-SF	Not specific		✓	2	✓			
Hewitt 2013 [33]	Australia	2013	RCT	Two-arm parallel			KDQoL-36			✓	2		✓		
Huang 2020 [34]	China	2020	RCT	Two-arm parallel			KDQoL-36		✓	✓	1				
Karkar 2015 [35]	Saudi Arabia	2015	Two-arm active treatments				KDQoL-SF			✓	2				✓
Khahi 2017 [36]	Iran	2017	RCT	Two-arm parallel			KDQoL-SF			✓	2	✓			
Lazarus 2018 [37]	India	2018	Quasi-RCT			✓	KDQoL-SF	Not specific	✓	✓	3	✓			
Liao 2020 [38]	China	2020	RCT	Two-arm parallel			KDQoL-36		✓	✓	2	✓			
Lim 2019 [39]	South Korea	2020	Quasi-RCT			✓	KDQoL-SF	Not specific	✓	✓	2	✓			
Macdougall [40]	UK	2019	RCT	Two-arm parallel	✓		KDQoL		✓	✓	5			✓	
Manfredini 2017 [41]	Italy	2017	RCT	Two-arm parallel	✓		KDQoL-SF		✓	✓	2		✓		
Manns 2009 [42]	Canada	2009	RCT	Two-arm parallel	✓		KDQoL-SF		✓	✓	2		✓		
Mansouri 2020 [43]	Iran	2020	Quasi-RCT			✓	KDQoL-SF	KDQoL total score	✓	✓	2	✓			
Martin-Alemany 2016 [44]	Mexico	2016	Quasi-RCT			✓	KDQoL-SF	Not specific	✓	✓	2	✓			
Martin-Alemany 2020 [45]	Mexico	2020	RCT	Multi-arm parallel			KDQoL-SF		✓	✓	2	✓			
Maslakpak 2015 [46]	Iran	2014	Quasi-RCT			✓	KDQoL-SF	KDQoL total score		✓	2	✓			
Mateti 2017 [47]	South India	2017	RCT	Two-arm parallel	✓	✓	KDQoL-36	Not specific	✓	✓	3			✓	
Maynard 2019 [48]	Brazil	2019	RCT	Two-arm parallel			KDQoL-SF		✓	✓	2	✓			
Medeiros 2019 [49]	Brazil	2018	Quasi-RCT				KDQoL-SF		✓	✓	2	✓			
Moeinzadeh 2016 [50]	Iran	2016	RCT	Two-arm parallel			KDQoL-SF			✓	2		✓		
Morais 2020 [51]	Brazil	2020	RCT	Two-arm parallel			KDQoL-SF		✓	✓	2	✓			
Morena 2017 [52]	France	2017	Quasi-RCT		✓		KDQoL-SF		✓	✓	4				✓
Naseri-Salahshour 2020 [53]	Iran	2020	RCT	Two-arm parallel		✓	KDQoL-SF	KDQoL total score	✓	✓	2	✓			
Oshvandi 2019 [54]	Iran	2018	Quasi-RCT			✓	KDQoL-SF	KDQoL total score		✓	2	✓			
Parsons 2006 [55]	Multi- country	2006	One group repeated measures		✓		KDQoL-SF			✓	1			✓	
Pellizzaro 2013 [56]	Brazil	2013	RCT	Two-arm parallel			KDQoL-SF			✓	1				
Poulsen 2017 [57]	Denmark	2017	RCT		✓		KDQoL-SF		✓	✓	3			✓	
Saglimbene 2008 [58]	Italy	2017	RCT	Two-arm parallel	✓		KDQoL-SF		✓	✓	3			✓	
Shahnavazi 2018 [59]	Iran	2017	Quasi-RCT			✓	KDQoL-SF	KDQoL total score	✓	✓	3	✓			
Sihombing 2017 [60]	Indonesia	2016	Quasi-RCT		✓		KDQoL-SF			✓	2		✓		
Singer 2011 [61]	Australia	2010	RCT	Two-arm parallel		✓	KDQoL-SF	KDQoL-SF symptom domain	✓	✓	2	✓			
Singer 2018 [62]	Australia	2019	RCT	Two-arm parallel			KDQoL-SF		✓	✓	4			✓	
Smith 2017 [63]	UK	2017	RCT	Crossover	✓		KDQoL-SF		✓	✓	2	✓			
Sofia 2013 [64]	Indonesia	2013	RCT	Two-arm parallel		✓	KDQoL-SF	Not specific	✓	✓	1				
Suhardjono 2019 [65]	Indonesia	2019	RCT	Multi-arm parallel			KDQoL-SF			✓	2	✓			
Tarverdizade 2016	Iran	2018	RCT	Multi-arm parallel			KDQoL-SF		✓	✓	2	✓			
Tawney 2000 [66]	USA	2000	RCT	Two-arm parallel	✓	✓	KDQoL-SF	KDQoL physical function domain	✓	✓	2		✓		
Uma 2016 [67]	India	2016	RCT	Two-arm parallel		✓	KDQoL-SF	KDQoL total score		✓	2	✓			
Wang 2008 [68]	Canada	2008	RCT	Crossover	✓	✓	KDQoL-SF	KDQoL symptom domain	✓	✓	4		✓		
Wang 2014 [69]	China	2014	RCT	Two-arm parallel		✓	KDQoL-SF	KCDS	✓	✓	4			✓	
Wu 2014 [70]	China	2014	RCT	Two-arm parallel		✓	KDQoL-SF	Not specific	✓	✓	2	✓			
Yuenyongchaiwat 2017 [71]	Thailand	2020	RCT	Two-arm parallel			KDQoL-36			✓	2	✓			
Zhang 2020 [72]	China	2020	RCT	Two-arm parallel			KDQoL-SF		✓	✓	1				
Zhang 2020 [73]	China	2020	RCT	Two-arm parallel			KDQoL-SF		✓	✓	2	✓			
Zheng 2019 [74]	China	2019	RCT	Two-arm parallel	✓		KDQoL-SF		✓	✓	2	✓			
Total					18	21			45	60		32	10	9	3


### KDQoL reporting

Table 3 presents how the individual trials reported the domains and summary scores for the KDQoL questionnaires. This table highlights the inconsistencies in reporting in current practice. Generally, trials do not use the kidney disease component summary (1) [75] or kidney summary score (0) [76] to summarize the kidney disease-specific domains. The summary scores from the SF-12/36, physical component score (PCS) and mental component score (MCS) were used by 40% (*n* = 24) of the trials.

**Table 3.** KDQoL reporting

	Domains^a^																			Summary scores^b^					
Study ID	Symptoms	Effects	Burden	Work status	Cognitive function	Social interaction	Sexual function	Sleep	Social support	Staff encouragement	Patient satisfaction	Physical functioning	Role physical	Pain	Genera health	Emotional well-being	Role emotional	Social function	Vitality	Physical	Mental	Kidney disease	Total score	VAS	Domains not explicit
Atevik 2020	✓	✓	✓																	✓	✓		✓		
Borzou 2020 [17]	✓	✓	✓	✓	✓	✓	✓	✓	✓	✓	✓	✓	✓	✓	✓	✓	✓	✓	✓						
Chang 2016 [18]	✓	✓	✓																	✓	✓				
Cukor 2014 [19]																							✓		
Dai 2020 [20]		✓	✓		✓	✓	✓		✓			✓		✓	✓								✓	✓	
de Lima 2013 [21]	✓						✓	✓	✓		✓	✓			✓				✓						
deFreitas 2020 [22]																				✓	✓				
Deziel 2007 [23]	✓	✓	✓	✓	✓	✓	✓	✓	✓	✓	✓	✓	✓	✓	✓	✓	✓	✓	✓					✓	
Duarte 2009 [24]	✓	✓	✓	✓	✓	✓	✓	✓	✓	✓	✓									✓	✓			✓	
Feldt-Rasmussen 2006 [25]	✓	✓	✓	✓	✓	✓	✓	✓	✓	✓	✓	✓	✓	✓	✓	✓	✓	✓	✓						
Figueiredo 2018 [26]	✓	✓	✓	✓	✓			✓	✓	✓	✓														
Fitschen 2017 [27]																		✓	✓						
Foley 2009 [28]	✓	✓	✓	✓	✓	✓		✓	✓	✓	✓	✓	✓	✓	✓		✓	✓	✓						
Fukuda 2015 [29]																									
Greenwood 2021 [30]												✓	✓	✓	✓		✓	✓		✓	✓				
Habibzadeh 2020 [31]																							✓		
Heo 2016 [32]	✓	✓	✓	✓	✓	✓	✓	✓	✓	✓	✓	✓	✓	✓	✓	✓	✓	✓	✓	✓	✓	✓		✓	
Hewitt 2013 [33]																									✓
Huang 2020 [34]	✓	✓	✓																	✓	✓				
Karkar 2015 [35]																									✓
Khahi 2017 [36]	✓	✓		✓	✓	✓	✓	✓	✓		✓	✓	✓	✓	✓		✓	✓							
Lazarus 2018 [37]	✓	✓	✓	✓	✓	✓	✓	✓	✓	✓	✓	✓	✓	✓	✓	✓	✓	✓	✓					✓	
Liao 2020 [38]	✓	✓	✓																	✓	✓				
Lim 2019 [39]	✓	✓	✓	✓	✓	✓	✓	✓	✓	✓	✓	✓	✓	✓	✓	✓	✓	✓	✓	✓	✓	✓	✓	✓	
Macdougall [40]																							✓		
Manfredini 2017 [41]	✓	✓	✓	✓	✓	✓	✓	✓	✓	✓	✓	✓	✓	✓	✓	✓	✓	✓	✓				✓		
Manns 2009 [42]	✓	✓	✓					✓				✓	✓	✓	✓		✓	✓	✓	✓	✓				
Mansouri 2020 [43]															✓								✓		
Martin-Alemany 2016 [44]	✓	✓	✓	✓	✓	✓	✓	✓	✓	✓	✓	✓	✓	✓	✓	✓	✓	✓	✓						
Martin-Alemany 2020 [45]	✓	✓	✓	✓	✓	✓	✓	✓	✓	✓	✓	✓	✓	✓	✓	✓	✓	✓	✓						
Maslakpak 2015 [46]												✓											✓		
Mateti 2017 [47]	✓	✓	✓									✓	✓	✓	✓	✓	✓	✓	✓						
Maynard 2019 [48]	✓	✓	✓	✓	✓	✓	✓	✓	✓	✓	✓	✓	✓	✓	✓	✓	✓	✓	✓	✓	✓				
Medeiros 2019 [49]	✓	✓	✓	✓	✓	✓	✓	✓	✓	✓	✓	✓	✓	✓	✓	✓	✓	✓	✓						
Moeinzadeh 2016 [50]																							✓		
Morais 2020 [51]	✓	✓	✓	✓	✓	✓	✓	✓	✓	✓	✓	✓	✓	✓	✓	✓	✓	✓	✓	✓	✓			✓	
Morena 2017 [52]			✓																	✓	✓				
Naseri-Salahshour 2020 [53]																							✓		
Oshvandi 2019 [54]																							✓		
Parsons 2006 [55]			✓								✓		✓			✓	✓	✓							
Pellizzaro 2013 [56]	✓							✓						✓					✓						
Poulsen 2017 [57]	✓	✓	✓	✓	✓	✓		✓	✓	✓	✓	✓	✓	✓	✓	✓	✓	✓	✓	✓	✓			✓	
Saglimbene 2008 [58]	✓	✓	✓	✓	✓	✓		✓	✓	✓	✓	✓	✓	✓	✓	✓	✓	✓	✓	✓	✓			✓	
Shahnavazi 2018 [59]	✓	✓		✓	✓	✓	✓	✓	✓	✓	✓	✓	✓	✓	✓		✓	✓					✓		
Sihombing 2017 [60]	✓	✓	✓	✓	✓	✓	✓	✓	✓	✓	✓	✓	✓	✓	✓	✓	✓	✓	✓						
Singer 2011 [61]	✓																								
Singer 2018 [62]	✓																								
Smith 2017 [63]																				✓	✓				
Sofia 2013 [64]	✓	✓	✓	✓	✓	✓	✓	✓	✓	✓	✓	✓	✓	✓	✓	✓	✓	✓	✓	✓	✓			✓	
Suhardjono 2019 [65]																				✓	✓				
Tarverdizade 2016																							✓		
Tawney 2000 [66]	✓	✓	✓	✓	✓	✓		✓	✓	✓	✓	✓	✓	✓	✓		✓	✓	✓	✓	✓				
Uma 2016 [67]	✓	✓	✓																	✓	✓		✓		
Wang 2008 [68]	✓	✓	✓	✓	✓	✓	✓	✓	✓	✓	✓	✓	✓	✓	✓	✓	✓	✓	✓						
Wang 2014 [69]	✓	✓	✓	✓	✓	✓	✓	✓	✓	✓	✓	✓	✓	✓	✓	✓	✓	✓	✓				✓		
Wu 2014 [70]				✓	✓	✓	✓	✓	✓	✓	✓	✓	✓	✓	✓	✓	✓	✓	✓						
Yuenyongchaiwat 2017 [71]	✓	✓	✓																	✓	✓				
Zhang 2020 [72]												✓	✓				✓		✓						
Zhang 2020 [73]	✓	✓	✓	✓	✓	✓	✓	✓	✓	✓	✓	✓	✓	✓	✓	✓	✓	✓	✓	✓	✓			✓	
Zheng 2019 [74]	✓	✓	✓	✓	✓	✓	✓	✓	✓	✓	✓	✓	✓	✓	✓	✓	✓	✓	✓	✓	✓				
Total	39	36	36	27	28	27	24	30	29	26	29	32	30	30	31	23	30	30	29	24	24	2	16	11	2

^a^A total of 19 health domains (detailed in Fig. 1).

^b^Summary scores reported in the literature (detailed in Fig. 1).

The number of trials generating a ‘KDQoL total score’ was 16 (27%). The methods used to calculate the total score are included in Supplementary data, Appendix Table A4. Most of these trials [11 (69%)] failed to explain the methods for calculating the total score. The authors of these trials (*n* = 11) were contacted for further information and one responded to the e-mail. Of the six trials for which we could determine the methods for calculating the total score: two took an average of the 19 domains; one took an average of the 11 kidney-specific domains; one took the median value of the domains; one summed the PCS, MCS, effects, burden and symptoms scores and one used a visual analogue scale (VAS) of overall health.

### Statistical analysis

We evaluated the statistical techniques used in the trials to make comparisons between treatment groups. This is detailed in Supplementary data, Appendix Table A4.

#### Between-group comparisons

The majority of trials [48 (79%)] did a between-group analysis that was unadjusted for other factors. A total of 11% of trials did not conduct a comparison between groups. This included the trial where between-group comparisons were not possible due to only considering repeated measures within a single group. Only 10% of trials adjusted for baseline covariates in the comparison between groups.

#### Within-group analysis

Almost half of trials conducted a within-group analysis [26 (41%)]. Most of these trials [17 (84%)] conducted their within-group analysis alongside a between-group analysis. The remaining studies [9 (16%)] only reported a within-group comparison.

### Missing data

**Figure 3:**
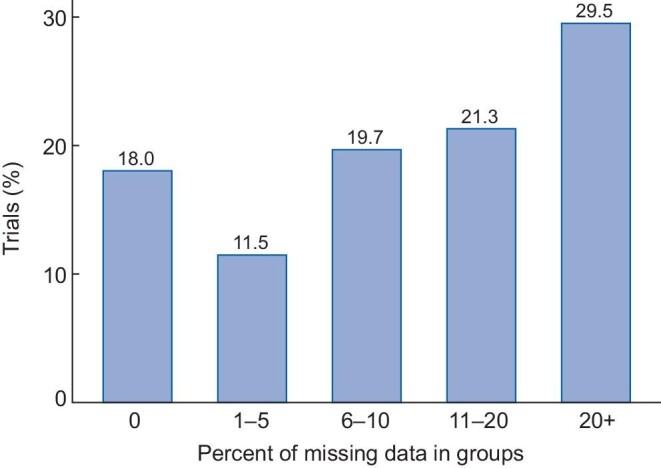
Percentage of dropouts relative to the number of patients randomized.

Details relating to missing data are provided in Table 4. The extent of missing data due to dropouts relative to the number of patients randomized is detailed in Fig. 3. Almost a third (30%) of trials had >20% of patients drop out, despite the majority [42 (69%)] of trials having a duration of <6 months.

**Table 4.** Missing data

Study ID	Patients randomized	Arms	Randomized—control group	Randomized—arm 1	Randomized—arm 2	Randomized—arm 3	Inflated sample size for dropout	Expected dropout (%)	Dropouts, *n* (%)	Dropouts/month of study	Methods for dealing with dropouts	Dropout death (*n*)	Death (%)	Dropout transplant (*n*)	Transplant (%)	Dropout other (*n*)	Other (%)	Sensitivity analysis	Dropout—adverse effects	Dropout—withdrawal of consent	Dropout—discontinuation of treatment
Atevik 2020	50	2	25	25			✓		0	0	No explicit missing data	0		0							
Borzou 2020 [17]	60	2	30	30					6 (10)	4	CCA	3	5	2	3	1	2				
Chang 2016 [18]	48	2	27	21					2 (4)	1	CCA	0		0		2	4		✓		
Cukor 2014 [19]	65	2	27	38			✓		6 (9)	2	CCA	0		1	2	5	8	✓	✓		
Dai 2020 [20]	140	2	70	70			✓	10	0	0	No explicit missing data	0		0							
de Lima 2013 [21]	33	3	11	11	11				1 (3)	2	CCA	0		0		1	3		✓		
deFreitas 2020 [22]	87	2	40	47					25 (29)	1	CCA	11	13	14	16	0			✓	✓	✓
Deziel 2007 [23]	57	2	28	29					13 (23)	2	CCA	6	11	2	4	5	9				✓
Duarte 2009 [24]	90	2	44	46					16 (18)	2	CCA	8	9	4	4	4	4		✓	✓	
Feldt-Rasmussen 2006 [25]	139	4	34	37	34	34			55 (40)		CCA	12	9	0		43	31		✓	✓	
Figueiredo 2018 [26]	37	3		11	13	13			6 (16)	3	CCA	2	5	1	3	3	8		✓	✓	
Fitschen 2017 [27]	41	2	21	20					8 (20)	1	CCA	3	7	0		5	12		✓	✓	
Foley 2009 [28]	596	2		300	296		✓	40	272 (46)	11	CCA	0		133	22	139	23		✓	✓	✓
Fukuda 2015 [29]	202	2	99	103					29 (14)	10	CCA	0		0		29	14		✓	✓	✓
Greenwood 2021 [30]	335	2	160	175					92 (27)	15	CCA	11	3	20	6	61	18	✓	✓	✓	✓
Habibzadeh 2020 [31]	120	4	30	30	30	30	✓		0	0	No explicit missing data	0		0							
Heo 2016 [32]	40	2	20	20					11(28)	11	CCA	0		0		11	28		✓	✓	✓
Hewitt 2013 [33]	60	2	30	30			✓	20	15(25)	3	CCA	2	3	2	3	11	18		✓		
Huang 2020 [34]	47	2	23	24			✓	10	19(40)		Single imputation	0		18	38	1	2		✓	✓	✓
Karkar 2015 [35]	72	2		36	36				0	0	No explicit missing data	0		0							
Khahi 2017 [36]	64	2	32	32			✓		9 (15)	5	CCA	0		3	5	6	9			✓	
Lazarus 2018 [37]	150	2	75	75					0	3	CCA	0		0							
Liao 2020 [38]	128	2	64	64					5(4)	0	No explicit missing data	0		0		5	4				
Lim 2019 [39]	50	2		25	25		✓	10	1(2)	2	CCA	0		0		1	2			✓	
Macdougall [40]	2141	2	1048	1093			✓	10	1246 (58)	0	CCA	515	24	371	17	360	17		✓	✓	✓
Manfredini 2017 [41]	296	2	145	151					69 (23)	104	CCA	5	2	7	2	57	19		✓	✓	
Manns 2009 [42]	52	2	25	27			✓	20	5 (10)	12	Single imputation	1	2	2	4	2	4	✓			✓
Mansouri 2020 [43]	64	2	32	32					4(6)	1	CCA	0		0		4	6		✓		✓
Martin-Alemany 2016 [44]	44	2		22	22				8 (18)	4	CCA	1	2	2	5	5	11				✓
Martin-Alemany 2020 [45]	45	3		15	15	15	✓	10	11(24)	3	CCA	1	2	6	13	4	9		✓		✓
Maslakpak 2015 [46]	120	3	40	40	40					4	CCA	0		0		0					
Mateti 2017 [47]	200	2	100	100			✓	20	47(24)	0	No explicit missing data	21	11	3	2	23	12				✓
Maynard 2019 [48]	45	2	23	22					5(11)	4	CCA	1	2	0		4	9		✓	✓	
Medeiros 2019 [49]	24	2	12	12			✓	20	3(13)	2	Single imputation	0		0		3	13	✓	✓	✓	✓
Moeinzadeh 2016 [50]	52	2	26	26					0	2	CCA	0		0							
Morais 2020 [51]	74	2	37	37			✓		13(18)	0	No explicit missing data	3	4	4	5	6	8			✓	✓
Morena 2017 [52]	381	2		191	190		✓	10	120(31)	9	CCA	79	21	7	2	34	9		✓	✓	✓
Naseri-Salahshour 2020 [53]	104	2	52	52					10(10)	5	Unclear	0		0		10	10			✓	✓
Oshvandi 2019 [54]	100	2	50	50					7(7)	5	CCA	2	2	4	4	1	1			✓	
Parsons 2006 [55]	20	1		20					7(35)	5	CCA	0		0		7	35		✓	✓	✓
Pellizzaro 2013 [56]	45	3							6(13)		CCA	1	2	0		5	11			✓	
Poulsen 2017 [57]	82	1		82					26(32)		CCA	3	4	7	9	16	20		✓	✓	✓
Saglimbene 2008 [58]	656	2		332	324		✓	5	37(6)	2	CCA	0		3		34	5		✓	✓	✓
Shahnavazi 2018 [59]	47	2	24	23					4(9)	3	CCA	0		0		4	9		✓	✓	✓
Sihombing 2017 [60]	113	2		74	39					1	CCA	0		0		0					
Singer 2011 [61]	100	2	51	49					2(2)	0	No explicit missing data	0		1	1	1	1			✓	
Singer 2018 [62]	70	2	32	36					13(19)	1	CCA	0		2	3	11	16		✓	✓	✓
Smith 2017 [63]	100	2		50	50		✓	18	5(5)	1	CCA	4	4	0		1	1		✓	✓	
Sofia 2013 [64]	36	2	18	18					0	3	CCA	0		0							
Suhardjono 2019 [65]	120	3	39	42	39				12(10)		No explicit missing data	2	2	0		10	8		✓		✓
Tarverdizade 2016	60	3	20	20	20		✓	30	0	4	CCA	0		0							
Tawney 2000 [66]	99	2	48	51					17(17)	0	No explicit missing data	4	4	0		13	13			✓	✓
Uma 2016 [67]	120	2	60	60						3	CCA	0		0		0					
Wang 2008 [68]	18	4							6(33)	0	CCA	0		0		6	33	✓	✓	✓	✓
Wang 2014 [69]	62	2	31	31			✓	20	4(6)	1	Multiple imputation	0		0		4	6			✓	✓
Wu 2014 [70]	69	2	35	34					4(6)	0	CCA	1	1	1	1	2	3			✓	✓
Yuenyongchaiwat 2017 [71]	50	2	25	25					5(10)	1	CCA	0		1	2	4	8				
Zhang 2020 [72]	74	2	37	37					22(30)		CCA	3	4	0		19	26			✓	✓
Zhang 2020 [73]	90	2	45	45			✓	10	3(3)	1	CCA	0		1	1	2	2		✓		✓
Zheng 2019 [74]	46	2	23	23					7(15)	2	CCA	2	4	0		5	11			✓	✓
Average	141	2	64	72	74	23		16	38(18)			12	6	10	7	19	9				
Total							22											5	31	35	31

Most trials [45 (74%)] included a Consolidated Standards of Reporting Trials (CONSORT) flow diagram detailing the reasons for patient dropout post-randomization. A total of 22 trials (36%) considered the possibility of dropouts and inflated the required sample size accordingly, although only 17 of these 22 stated explicitly by how much. The expected dropout for these trials ranged from 5 to 40%, [interquartile range (IQR) 10–20)], but did not seem to be related to the duration of follow-up. Four trials (7%) mentioned that the high dropout rate may cause bias and could limit the interpretation of results.

#### Methods for dealing with missing QoL data (primary data analysis)

A total of 11 trials (18%) reported no missing data between randomization and the study endpoint, 45 (74%) used complete case analysis to deal with missing data, 4 (7%) used imputation and 1 (2%) was unclear on the methods and did not respond to e-mail. Three trials (5%) used single imputation methods. Single imputation methods included one trial carrying forward the baseline QoL data, while the other two trials carried forward the last observation. One trial used multiple imputation by using propensity methods to replace missing QoL values. Only one trial explicitly mentioned the missing data mechanism assumption when justifying the methods for dealing with dropouts.

#### Sensitivity analysis relating to missing QoL data

Sensitivity analysis relating to missing QoL data was conducted by five trials (7%). Four trials (7%) conducted either complete case analysis or single imputation for their sensitivity analysis. The fifth trial performed two types of sensitivity analyses: imputing patients who died with a value of 0 and performing multiple imputation. All trials concluded that the sensitivity analysis did not change the interpretation of the results.

#### Deaths

A total of 27 trials (44%) recorded dropout due to death, with the extent of dropouts ranging from 1 to 24% of the total number of patients randomized [median 4% (IQR 2–8)]. The only death-specific imputation found in this review was one trial that imputed QoL values to zero for patients who died.

#### Transplants

A total of 28 trials (46%) recorded dropout due to transplants, with the extent of dropouts ranging from 1 to 38% of the total number of patients randomized [median 4% (IQR 2–8)]. No transplant-specific imputation analyses were found when reviewing these trials.

## DISCUSSION

The aim of this review was to explore how current nephrology trials use, report and analyse the KDQoL questionnaires when evaluating QoL in patients receiving HD treatment. The review identified a number of methodological issues, including amending validated versions of the questionnaires against the recommendations of the developers, reporting a KDQoL total score, reporting only statistically significant results, failing to account for missing QoL data appropriately (specifically death/transplant data) and using limited methods in the statistical analysis of trials. The above methodological issues may be biasing the results of these trials and contributing to the limited number of nephrology trials concluding with positive results and therefore impacting clinical practice. This, in turn, could be limiting the opportunity for improvements in the QoL of the HD population. These findings support previous literature relating to the poor methodological quality of nephrology trials [4]. However, this is the first article to examine the reporting quality of KDQoL and explore the methods used in the primary data analysis to account for dropouts, especially due to death and transplant.

### KDQoL reporting and analysis

This review identified inconsistencies in how trials reported the results of the KDQoL questionnaires, including generating a single index of QoL, which is not recommended by the developers of the KDQoL questionnaires due to the multidimensional nature of the tool [7]. The misuse of a KDQoL total score is a common issue among users of the SF-36. A review of the use of the SF-36 total score [77] found 172 articles calculating a total score as a single measure of health, against the recommendations of the developers. In line with our findings, many [129 (75%)] were unclear on the methods used to calculate the total score. The KDQoL developers emphasize the need to analyse physical and mental health domains separately, similar to the recommendations for the SF-12/36 [78]. Researchers were also found to have modified the standardized KDQoL questionnaire, excluding certain domains due to the focus of the trial (e.g. fatigue) or sensitivity of the questions (e.g. sexual function), and/or reported only those domains that were statistically significant in their trial publications. The tendency to report only significant domains is a form of reporting bias, suggesting that some authors may be cherry-picking significant results and presenting these as the main results to emphasize their findings.

### Appropriate use of statistical methods

In this review, trials reporting the KDQoL as their primary outcome did not explicitly specify which component of the KDQoL formed their primary outcome. These trials referred to multiple domains when reporting the effectiveness of the interventions, making it unclear to readers the focus of the trials. Lack of clarity in the primary outcome can also lead to questions about the sufficiency of the sample size and power of the trial. Generally these trials provided vague explanations of their sample size calculations or omitted this information completely. Many trials conducted within-group statistical comparisons, comparing measurements at baseline and follow-up, which have been widely reported to be invalid and produce conclusions that are potentially misleading [79]. As well as this, only a few trials adjusted for baseline covariates, which generally improve the efficiency of the analysis, leading to a substantial increase in power [80]. Trials used linear models and linear mixed models to analyse the longitudinal evolution of health-related quality of life (HRQoL), which are valid when MCAR and MAR assumptions are met. Despite this, few trials discussed whether dropouts were MNAR, MAR or MCAR, and 75% of trials had at least one dropout due to transplants, adverse effects or death. Therefore it is likely these models were used in invalid conditions, which increases the potential risk of bias.

### Missing data

Missing data were a common occurrence in the trials reviewed. The potential bias due to missing data depends on the reason for the missingness. Complete case analysis and single imputation methods assume that missing data are MCAR, meaning the reason for dropout is unrelated to the intervention or disease. However, in these trials, missing data were commonly due to death, transplant, ill health or treatment switching. This means that the dropout was likely related to the intervention or disease and therefore not MCAR. Few trials performed sensitivity analysis to assess the impact of the missing data assumptions on the results or discussed the potential bias due to missing data.

Similar investigations into missing data in other populations have found that complete case analysis and single imputation methods are widely used for dealing with dropouts in clinical trials. Thabut *et al*. [81] conducted a review of missing data in 16 idiopathic pulmonary fibrosis trials: 50% (*n* = 8/16) of trials conducted complete case analysis, 31% (*n* = 5/16) conducted last observation carried forward and the remaining trials conducted various single imputation methods. Hamel *et al*. [13] conducted a review of the methodological quality of cancer trials when analysing QoL data. A total of 33 trials were included in this review and 94% (31/33) of trials conducted complete case analysis to deal with missing QoL data. It seems sensible to conclude that missing data due to dropouts are poorly dealt with across many medical specialities and more robust statistical techniques are needed to account for these events in clinical trials.

### Strengths and limitations

The search strategy for this review was developed and reviewed by a consultant nephrologist and a health sciences librarian. This work is based on published trials available in English and may therefore be subject to publication and language bias. However, as this is a method-based review, it is not anticipated that this will have a significant impact on the results. It has been reported that language restrictions do not lead to evidence of systematic bias in review-based analyses [82]. Several trials (*n* = 14) did not have sufficient information in their articles to populate the extraction form. These authors were contacted but very few responded (*n* = 1). However, we believe the included data provide sufficient evidence to make conclusions on the current practice of nephrology trials relating to the handling of dropouts and KDQoL reporting. Our study also adhered to the PRISMA reporting guidelines.

### Implications for future research

This review highlights the lack of implementation of appropriate methods when dealing with dropouts in clinical trials and the inconsistencies in reporting the validated KDQoL questionnaires. There is currently no consensus on dealing with dropouts due to death, transplantation and ill health, which are common causes of attrition in the HD population. There is an urgent need for nephrology trials to become more methodologically coherent. Poor reporting and inappropriate analyses of QoL data lead to uncertainty over which treatments may have a significant impact on the QoL of patients receiving HD. By addressing these methodological limitations, the quality of clinical trials within the field of nephrology will be enhanced, increasing their ability to influence clinical practice for the benefit of people receiving HD and their families.

## CONCLUSIONS

Inadequate reporting and handling of missing QoL data in RCTs still exists. It appears that there exists a large gap between statistical methods for dealing with missing data and their application in practice. This work forms the basis for future guidance on addressing missing QoL data in clinical trials. This review focused on nephrology trials, which have a unique form of dropout due to transplantation, but it is intended that future method development and guidelines apply to any setting where QoL data are collected. It also highlights the inconsistency of reporting the KDQoL, the failure of reporting a primary outcome measure, cherry-picking results and altering validated questionnaires; these are statistical issues that researchers should avoid. Journals must enforce good practice to ensure a higher standard of research. Better, more robust reporting will further identify treatments that could improve QoL within the HD population.

## Supplementary Material

gfac170_Supplemental_File
