# Supplementary material for: Measuring quality of life in trials including patients on haemodialysis: methodological issues surrounding the use of the Kidney Disease Quality of Life Questionnaire
Source: Nephrol Dial Transplant. 2022 Jun 11;37(12):2538–54. doi: 10.1093/ndt/gfac170 (PMC9681926; doi:10.1093/ndt/gfac170)
Supplement: gfac170_Supplemental_File [file gfac170_supplemental_file.docx]

**Tables Appendix**

**Table A1: Advantages and disadvantages of methods used to analyse missing HRQoL data**

| *Method* | *Description* | *Advantage* | *Disadvantage* |
| --- | --- | --- | --- |
| Complete case analysis | Excludes any patient with a missing HRQoL value | Easy to implement  Simple | Invalid under MNAR.  Does not make use of the information collected about these participants. |
| Last observation carried forward | Imputes the missing value with the last recorded HRQoL value | Easy to implement  Simple | Invalid under MNAR.  Assumes HRQoL is fixed between time points. |
| Baseline observation carried forward | Imputes the missing value with the baseline recorded HRQoL value | Easy to implement  Simple | Invalid under MNAR.  Assumes HRQoL is fixed between time points. |
| Multiple Imputation | Missing values are imputed many times and are then consolidated into one result. | Estimates a mean, variance and confidence interval to account for uncertainty | Valid under MCAR or MAR |
| HRQoL health related quality of life, MNAR missing not at random, MCAR missing completely at random, MAR missing at random. | | | |

**Table A2: PRISMA Checklist**

| **Section and Topic** | **Item #** | **Checklist item** | **Location where item is reported** |
| --- | --- | --- | --- |
| **TITLE** | | |  |
| Title | 1 | Identify the report as a systematic review. | 1 |
| **ABSTRACT** | | |  |
| Abstract | 2 | See the PRISMA 2020 for Abstracts checklist. | 3 |
| **INTRODUCTION** | | |  |
| Rationale | 3 | Describe the rationale for the review in the context of existing knowledge. | 4 |
| Objectives | 4 | Provide an explicit statement of the objective(s) or question(s) the review addresses. | 4 |
| **METHODS** | | |  |
| Eligibility criteria | 5 | Specify the inclusion and exclusion criteria for the review and how studies were grouped for the syntheses. | 5 |
| Information sources | 6 | Specify all databases, registers, websites, organisations, reference lists and other sources searched or consulted to identify studies. Specify the date when each source was last searched or consulted. | 5 |
| Search strategy | 7 | Present the full search strategies for all databases, registers and websites, including any filters and limits used. | 34 |
| Selection process | 8 | Specify the methods used to decide whether a study met the inclusion criteria of the review, including how many reviewers screened each record and each report retrieved, whether they worked independently, and if applicable, details of automation tools used in the process. | 5 |
| Data collection process | 9 | Specify the methods used to collect data from reports, including how many reviewers collected data from each report, whether they worked independently, any processes for obtaining or confirming data from study investigators, and if applicable, details of automation tools used in the process. | 5 |
| Data items | 10a | List and define all outcomes for which data were sought. Specify whether all results that were compatible with each outcome domain in each study were sought (e.g. for all measures, time points, analyses), and if not, the methods used to decide which results to collect. | 5/6 |
|  | 10b | List and define all other variables for which data were sought (e.g. participant and intervention characteristics, funding sources). Describe any assumptions made about any missing or unclear information. | N/A |
| Study risk of bias assessment | 11 | Specify the methods used to assess risk of bias in the included studies, including details of the tool(s) used, how many reviewers assessed each study and whether they worked independently, and if applicable, details of automation tools used in the process. | N/A |
| Effect measures | 12 | Specify for each outcome the effect measure(s) (e.g. risk ratio, mean difference) used in the synthesis or presentation of results. | N/A |
| Synthesis methods | 13a | Describe the processes used to decide which studies were eligible for each synthesis (e.g. tabulating the study intervention characteristics and comparing against the planned groups for each synthesis (item #5)). | 6 |
|  | 13b | Describe any methods required to prepare the data for presentation or synthesis, such as handling of missing summary statistics, or data conversions. | N/A |
|  | 13c | Describe any methods used to tabulate or visually display results of individual studies and syntheses. | 6 |
|  | 13d | Describe any methods used to synthesize results and provide a rationale for the choice(s). If meta-analysis was performed, describe the model(s), method(s) to identify the presence and extent of statistical heterogeneity, and software package(s) used. | 6 |
|  | 13e | Describe any methods used to explore possible causes of heterogeneity among study results (e.g. subgroup analysis, meta-regression). | N/A |
|  | 13f | Describe any sensitivity analyses conducted to assess robustness of the synthesized results. | N/A |
| Reporting bias assessment | 14 | Describe any methods used to assess risk of bias due to missing results in a synthesis (arising from reporting biases). | N/A |
| Certainty assessment | 15 | Describe any methods used to assess certainty (or confidence) in the body of evidence for an outcome. | N/A |
| **RESULTS** | | |  |
| Study selection | 16a | Describe the results of the search and selection process, from the number of records identified in the search to the number of studies included in the review, ideally using a flow diagram. | 7 |
|  | 16b | Cite studies that might appear to meet the inclusion criteria, but which were excluded, and explain why they were excluded. | N/A |
| Study characteristics | 17 | Cite each included study and present its characteristics. | 8 |
| Risk of bias in studies | 18 | Present assessments of risk of bias for each included study. | N/A |
| Results of individual studies | 19 | For all outcomes, present, for each study: (a) summary statistics for each group (where appropriate) and (b) an effect estimate and its precision (e.g. confidence/credible interval), ideally using structured tables or plots. | N/A |
| Results of syntheses | 20a | For each synthesis, briefly summarise the characteristics and risk of bias among contributing studies. | 8/9/10 |
|  | 20b | Present results of all statistical syntheses conducted. If meta-analysis was done, present for each the summary estimate and its precision (e.g. confidence/credible interval) and measures of statistical heterogeneity. If comparing groups, describe the direction of the effect. | N/A |
|  | 20c | Present results of all investigations of possible causes of heterogeneity among study results. | N/A |
|  | 20d | Present results of all sensitivity analyses conducted to assess the robustness of the synthesized results. | N/A |
| Reporting biases | 21 | Present assessments of risk of bias due to missing results (arising from reporting biases) for each synthesis assessed. | N/A |
| Certainty of evidence | 22 | Present assessments of certainty (or confidence) in the body of evidence for each outcome assessed. | N/A |
| **DISCUSSION** | | |  |
| Discussion | 23a | Provide a general interpretation of the results in the context of other evidence. | 24/25/26 |
|  | 23b | Discuss any limitations of the evidence included in the review. | 24/25/26 |
|  | 23c | Discuss any limitations of the review processes used. | 24/25/26 |
|  | 23d | Discuss implications of the results for practice, policy, and future research. | 24/25/26 |
| **OTHER INFORMATION** | | |  |
| Registration and protocol | 24a | Provide registration information for the review, including register name and registration number, or state that the review was not registered. | 3 |
|  | 24b | Indicate where the review protocol can be accessed, or state that a protocol was not prepared. | 5 |
|  | 24c | Describe and explain any amendments to information provided at registration or in the protocol. | 6 |
| Support | 25 | Describe sources of financial or non-financial support for the review, and the role of the funders or sponsors in the review. | 2 |
| Competing interests | 26 | Declare any competing interests of review authors. | End |
| Availability of data, code and other materials | 27 | Report which of the following are publicly available and where they can be found: template data collection forms; data extracted from included studies; data used for all analyses; analytic code; any other materials used in the review. | End |

*From:*  Page MJ, McKenzie JE, Bossuyt PM, Boutron I, Hoffmann TC, Mulrow CD, et al. The PRISMA 2020 statement: an updated guideline for reporting systematic reviews. BMJ 2021;372:n71. doi: 10.1136/bmj.n71

For more information, visit: <http://www.prisma-statement.org/>

**Table A3: Medline Search Strategy**

Database: Medline 1946-date (includes epub ahead of print and in process citations. Updated daily)

Search Strategy:

--------------------------------------------------------------------------------

1 Dialysis/ or dialysis.mp. (171204)

2 dialyses.mp. (821)

3 dialysate.mp. or Dialysis Solutions/ (13351)

4 dialysates.mp. (1416)

5 dialyzate.mp. (165)

6 dialyzates.mp. (28)

7 microdialysis.mp. or Microdialysis/ (17875)

8 microdialyses.mp. (4)

9 kidney replacement therapy.mp. (301)

10 kidney replacement therapies.mp. (27)

11 renal replacement therapy.mp. or Renal Replacement Therapy/ (15443)

12 renal replacement therapies.mp. (821)

13 artificial kidney*.mp. or Kidneys, Artificial/ (4751)

14 blood dialyser*.mp. (4)

15 blood dialyzer*.mp. (22)

16 Renal Dialysis/ (92294)

17 CAPD.mp. or Peritoneal Dialysis, Continuous Ambulatory/ (11179)

18 *dialysis/ (2279)

19 *dialyses/ (2279)

20 Hemofiltration/ or hemofiltration*.mp. (6341)

21 haemofiltration*.mp. (961)

22 ultrafilteration*.mp. (5)

23 biofilteration*.mp. (2)

24 1 or 2 or 3 or 4 or 5 or 6 or 7 or 8 or 9 or 10 or 11 or 12 or 13 or 14 or 15 or 16 or 17 or 18 or 19 or 20 or 21 or 22 or 23 (202167)

25 quality of life.mp. or "Quality of Life"/ (357611)

26 Qol.mp. (40312)

27 Hql.mp. (99)

28 Hqol.mp. (110)

29 H qol.mp. (8)

30 Hrqol.mp. (17377)

31 Hr qol.mp. (827)

32 life quality.mp. (7693)

33 value of life.mp. or "Value of Life"/ (6115)

34 quality adjusted life year*.mp. or Quality-Adjusted Life Years/ (18974)

35 QALY.mp. (9183)

36 Disability adjusted life year*.mp. (3584)

37 DALY.mp. (1935)

38 kidney disease quality of life-SF.mp. (5)

39 kidney disease quality of life.mp. (394)

40 kidney disease quality of life-36.mp. (47)

41 KDQOL*.mp. (350)

42 sf6.mp. (1658)

43 sf 6.mp. (476)

44 short form 6.mp. (111)

45 sf six.mp. (5)

46 short form six.mp. (56)

47 6-item short form.mp. (31)

48 six item short form.mp. (47)

49 sf12.mp. (410)

50 sf 12.mp. (4650)

51 short form 12.mp. (2187)

52 shortform 12.mp. (1)

53 sf twelve.mp. (1)

54 12-item short form.mp. (1245)

55 sf16.mp. (8)

56 sf 16.mp. (21)

57 short form 16.mp. (4)

58 sf sixteen.mp. (1)

59 short form sixteen.mp. (1)

60 16-item short form.mp. (13)

61 sf20.mp. (39)

62 sf 20.mp. (298)

63 short form 20.mp. (83)

64 sf twenty.mp. (7)

65 short form twenty.mp. (1)

66 20-item short form.mp. (31)

67 sf36.mp. (1313)

68 sf 36.mp. (21025)

69 short form 36.mp. (10446)

70 shortform 36.mp. (13)

71 sf thirty six.mp. (1)

72 short form thirty six.mp. (1)

73 36-item short form.mp. (4489)

74 SF-36.mp. (21025)

75 SF-6D.mp. (798)

76 SF-6.mp. (476)

77 SF-12.mp. (4650)

78 SF-20.mp. (298)

79 SF-16.mp. (21)

80 25 or 26 or 27 or 28 or 29 or 30 or 31 or 32 or 33 or 34 or 35 or 36 or 37 or 38 or 39 or 40 or 41 or 42 or 43 or 44 or 45 or 46 or 47 or 48 or 49 or 50 or 51 or 52 or 53 or 54 or 55 or 56 or 57 or 58 or 59 or 60 or 61 or 62 or 63 or 64 or 65 or 66 or 67 or 68 or 69 or 70 or 71 or 72 or 73 or 74 or 75 or 76 or 77 or 78 or 79 (382375)

81 24 and 80 (6724)

82 Randomized Controlled Trials as Topic/ (138501)

83 Clinical Trials as Topic/ (193802)

84 trial*.mp. (1756963)

85 clinical trial.mp. or Clinical Trial/ (721882)

86 randomi?ed control* trial.mp. (560831)

87 non randomised study.mp. (411)

88 randomised study.mp. (4303)

89 non randomized study.mp. (1307)

90 randomized study.mp. (26638)

91 82 or 83 or 84 or 85 or 86 or 87 or 88 or 89 or 90 (1762534)

92 81 and 91 (1251)

N=1,251

**Table A4: KDQoL Analysis**

| Study ID | Methods total score | Unadjusted for other factors comparison of groups | Baseline adjusted comparison of groups | No between group analysis | Within group analysis |
| --- | --- | --- | --- | --- | --- |
| Atevik 2020 | Median | 🗸 |  |  | 🗸 |
| Borzou 2020^(17)^ |  | 🗸 |  |  | 🗸 |
| Chang 2016^(18)^ |  |  | 🗸 |  |  |
| Cukor 2014^(19)^ | Unclear | 🗸 |  |  |  |
| Dai 2020^(20)^ | Unclear | 🗸 |  |  | 🗸 |
| de Lima 2013^(21)^ |  | 🗸 |  | 🗸 |  |
| deFreitas 2020^(22)^ |  | 🗸 |  | 🗸 |  |
| Deziel 2007^(23)^ |  | 🗸 |  |  |  |
| Duarte 2009^(24)^ |  | 🗸 |  |  |  |
| Feldt-Rasmussen 2006^(25)^ |  |  | 🗸 |  |  |
| Figueiredo 2018^(26)^ |  | 🗸 |  |  |  |
| Fitschen 2017^(27)^ |  | 🗸 |  |  |  |
| Foley 2009^(28)^ |  |  |  |  |  |
| Fukuda 2015^(29)^ |  | 🗸 |  |  |  |
| Greenwood 2021^(30)^ |  |  | 🗸 |  |  |
| Habibzadeh 2020^(31)^ | Arithmetic average: Unclear | 🗸 |  |  | 🗸 |
| Heo 2016^(32)^ |  |  | 🗸 |  | 🗸 |
| Hewitt 2013^(33)^ |  | 🗸 |  |  |  |
| Huang 2020^(34)^ |  | 🗸 |  |  | 🗸 |
| Karkar 2015^(35)^ |  | 🗸 |  |  |  |
| Khahi 2017^(36)^ |  | 🗸 |  |  | 🗸 |
| Lazarus 2018^(37)^ |  | 🗸 |  |  |  |
| Liao 2020^(38)^ |  | 🗸 |  |  | 🗸 |
| Lim 2019^(39)^ | Arithmetic average: Unclear | 🗸 |  |  |  |
| Macdougall^(40)^ | Sum: PCS, MCS, Effects, Burden, Symptoms | 🗸 |  |  |  |
| Manfredini 2017^(41)^ | VAS overall measure of health | 🗸 |  |  | 🗸 |
| Manns 2009^(42)^ |  | 🗸 |  |  |  |
| Mansouri 2020^(43)^ | Unclear, but refers to developers recommendation (Hays et al) | 🗸 |  |  |  |
| Martin-Alemany 2016^(44)^ |  | 🗸 |  |  |  |
| Martin-Alemany 2020^(45)^ |  | 🗸 |  | 🗸 |  |
| Maslakpak 2015^(46)^ | Arithmetic average: Unclear | 🗸 |  |  |  |
| Mateti 2017^(47)^ |  | 🗸 |  |  |  |
| Maynard 2019^(48)^ |  | 🗸 |  |  | 🗸 |
| Medeiros 2019^(49)^ |  |  |  | 🗸 | 🗸 |
| Moeinzadeh 2016^(50)^ | Arithmetic average: Unclear | 🗸 |  |  | 🗸 |
| Morais 2020^(51)^ |  | 🗸 |  |  | 🗸 |
| Morena 2017^(52)^ |  |  |  |  |  |
| Naseri-Salahshour 2020^(53)^ | Arithmetic average: Unclear | 🗸 |  |  | 🗸 |
| Oshvandi 2019^(54)^ | Arithmetic average: Unclear | 🗸 |  |  | 🗸 |
| Parsons 2006^(55)^ |  |  |  | 🗸 | 🗸 |
| Pellizzaro 2013^(56)^ |  | 🗸 |  |  |  |
| Poulsen 2017^(57)^ |  | 🗸 |  |  | 🗸 |
| Saglimbene 2008^(58)^ |  | 🗸 |  |  |  |
| Shahnavazi 2018^(59)^ | Arithmetic average: 19 domains | 🗸 |  |  |  |
| Sihombing 2017^(60)^ |  | 🗸 |  |  |  |
| Singer 2011^(61)^ |  | 🗸 |  |  |  |
| Singer 2018^(62)^ |  | 🗸 |  |  | 🗸 |
| Smith 2017^(63)^ |  | 🗸 |  |  | 🗸 |
| Sofia 2013^(64)^ |  |  |  | 🗸 | 🗸 |
| Suhardjono 2019^(65)^ |  | 🗸 |  |  |  |
| Tarverdizade  2016 | Arithmetic average: 19 domains | 🗸 |  |  | 🗸 |
| Tawney 2000^(66)^ |  |  | 🗸 |  | 🗸 |
| Uma 2016^(67)^ | Arithmetic average: Unclear |  |  | 🗸 | 🗸 |
| Wang 2008^(68)^ |  |  |  |  |  |
| Wang 2014^(69)^ | Arithmetic average: 11 kidney disease domains |  | 🗸 |  |  |
| Wu 2014^(70)^ |  | 🗸 |  | 🗸 |  |
| Yuenyongchaiwat 2017^(71)^ |  | 🗸 |  |  |  |
| Zhang 2020^(72)^ |  | 🗸 |  |  | 🗸 |
| Zhang 2020^(73)^ |  |  |  | 🗸 | 🗸 |
| Zheng 2019^(74)^ |  | 🗸 |  |  |  |
| Total |  | 47 | 6 | 9 | 26 |
| ^VAS = visual analogue scale, PCS = Physical component summary, MCS = Mental component summary^ | | | | | |
